# Chimeric Newcastle Disease Virus Protects Chickens against Avian Influenza in the Presence of Maternally Derived NDV Immunity

**DOI:** 10.1371/journal.pone.0072530

**Published:** 2013-09-04

**Authors:** Constanze Steglich, Christian Grund, Kristina Ramp, Angele Breithaupt, Dirk Höper, Günther Keil, Jutta Veits, Mario Ziller, Harald Granzow, Thomas C. Mettenleiter, Angela Römer-Oberdörfer

**Affiliations:** 1 Institute of Molecular Biology, Friedrich-Loeffler-Institut, Federal Research Institute for Animal Health, Greifswald-Insel Riems, Germany; 2 Institute of Diagnostic Virology, Friedrich-Loeffler-Institut, Federal Research Institute for Animal Health, Greifswald-Insel Riems, Germany; 3 Department of Experimental Animal Facilities and Biorisk Management, Friedrich-Loeffler-Institut, Federal Research Institute for Animal Health, Greifswald-Insel Riems, Germany; 4 AG Bioinformatics, Friedrich-Loeffler-Institut, Federal Research Institute for Animal Health, Greifswald-Insel Riems, Germany; 5 Institute of Infectology, Friedrich-Loeffler-Institut, Federal Research Institute for Animal Health, Greifswald-Insel Riems, Germany; Kantonal Hospital St. Gallen, Switzerland

## Abstract

Newcastle disease virus (NDV), an avian paramyxovirus type 1, is a promising vector for expression of heterologous proteins from a variety of unrelated viruses including highly pathogenic avian influenza virus (HPAIV). However, pre-existing NDV antibodies may impair vector virus replication, resulting in an inefficient immune response against the foreign antigen. A chimeric NDV-based vector with functional surface glycoproteins unrelated to NDV could overcome this problem. Therefore, an NDV vector was constructed which carries the fusion (F) and hemagglutinin-neuraminidase (HN) proteins of avian paramyxovirus type 8 (APMV-8) instead of the corresponding NDV proteins in an NDV backbone derived from the lentogenic NDV Clone 30 and a gene expressing HPAIV H5 inserted between the F and HN genes. After successful virus rescue by reverse genetics, the resulting chNDVFHN _PMV8_H5 was characterized *in vitro* and *in vivo*. Expression and virion incorporation of the heterologous proteins was verified by Western blot and electron microscopy. Replication of the newly generated recombinant virus was comparable to parental NDV in embryonated chicken eggs. Immunization with chNDVFHN _PMV8_H5 stimulated full protection against lethal HPAIV infection in chickens without as well as with maternally derived NDV antibodies. Thus, tailored NDV vector vaccines can be provided for use in the presence or absence of routine NDV vaccination.

## Introduction

Highly pathogenic avian influenza viruses (HPAIV) are a constant risk for poultry throughout the world. 7353 outbreaks of HPAI H5N1 in poultry in 52 countries have been reported since 2003 [1], most of them in Vietnam (2,681), Thailand (1,141), and Egypt (1,084). These outbreaks are accompanied by infections of humans with 630 reported cases, including 375 deaths, primarily in Indonesia, Egypt and Vietnam [2]. In combination with epidemiological surveillance and enforcement of biosafety measures, vaccination is a proven method to control HPAIV. Vaccines against HPAIV are mostly inactivated, oil adjuvanted virus preparations which are licensed in many countries [3]. However, these vaccines are problematic, because of their propagation in embryonated chicken eggs (ECE) requiring handling a highly virulent pathogen in great amounts under frequently less than ideal conditions. Moreover, HPAIV replication in ECE often leads to an early death of chicken embryos resulting in low virus yield and high costs. Therefore, there is an urgent need to develop an HPAIV vaccine which combines safety, low production costs and high efficiency. The development of attenuated replication-competent vector vaccines is highly promising in this respect. Different avian viruses like fowlpox virus [4], infectious laryngotracheitis virus [5], herpesvirus of turkey [6], and NDV [7–10] had been developed into vector viruses for the expression of heterologous proteins including HPAIV antigens.

Recombinant NDV expressing among others the major HPAIV antigen hemagglutinin (HA), were constructed by reverse genetics [7–11]. Immunization of specific pathogen free (SPF) chickens with these viruses protected from lethal HPAIV infection [7,8,12,13], resulting in their field use, e.g. in China and Mexico [14,15]. However, the performance was not satisfactory. Importantly, commercial chickens frequently carry maternally derived NDV antibodies due to vaccination or natural infection, which may result in neutralization of the vector virus and a low immune response against the heterologous antigen [16]. An NDV-based vector chimeric by functional non-NDV surface proteins could overcome this problem.

Avian Paramyxovirus-1 (APMV-1) including NDV, and APMV-2 to -9 are members of the genus *Avulavirus* in the *Paramyxoviridae* family [17]. Recently, further APMVs have been isolated and were described as APMV-10, -11, and -12 [18–20]. The APMV genomes consist of non-segmented, negative stranded RNA of between about 15 kb and about 17 kb [19,21]. The number of nucleotides in the genome represents a multiple of six following the “rule of six” for efficient replication as shown for NDV and other *paramyxoviruses* [22,23]. The six protein encoding genes (except for APMV-6 which carries seven genes) are surrounded by a 3’-leader- and a 5’-trailer sequence and are individually flanked by conserved gene start and gene end sequences [21]. The close phylogenetic relationship of APMV-2 to -9 and NDV with simultaneous differences in antigenicity opens up the possibility to substitute the surface proteins of NDV by those of another APMV to generate a virus which could overcome impairment by maternally derived NDV antibodies. Similar chimeric or pseudotyped viruses have already been described e.g. for vesicular stomatitis virus [24–26] and rabies virus [27]. NDV with an F protein that was substituted by that of APMV-2 or surface proteins consisting of parts of NDV and APMV-2 polypeptides have been isolated [28]. Furthermore, a recombinant NDV with a chimeric HN encompassing the cytoplasmic domain, the transmembrane region, and the stalk region of NDV and the immunogenic globular head of APMV-4 was successfully generated [29]. These viruses also allowed serological differentiation between vaccine and field virus. However, the creation of a chimeric NDV expressing three heterologous proteins, two derived from APMV and one from HPAIV, has not been reported before.

Here, we describe the construction of such a chimeric NDV functionally replacing the NDV surface glycoproteins F and HN by those of APMV-8 and expressing, in addition, HPAIV H5N1 HA. APMV-8 was selected as donor because of low serological cross reactivity between APMV-8 and NDV [30,31]. The resulting prototypic vaccine virus was able to protect SPF chickens from a lethal infection with HPAIV H5N1 in the absence and presence of maternally derived NDV antibodies.

## Materials and Methods

### Cells and viruses

Chicken embryo fibroblasts (CEF) prepared from 10-day-old SPF embryonated chicken eggs (ECE), and quail muscle cells clone 9 (QM9) were used for *in vitro* investigations. BSR-T7 cells [32] which stably express phage T7 RNA polymerase, were used to recover infectious virus from cDNA. Virus titration of swabs taken after vaccination and challenge during the animal experiment was done on Leghorn hepatocellular epithelial cells (LMH). ECE for virus propagation and cell preparation were purchased from Lohmann, Cuxhaven, Germany and incubated at 37 °C and 55% humidity.

Recombinant NDV (rNDVGu) on the basis of NDV Clone 30, and NDVH5Vm have already been described [33,34]. HPAIV A/duck/Vietnam/TG24-01/05 (H5N1) (Clade: 1) was kindly provided by P. Song Lien (National Centre for Veterinary Diagnosis, Dongda, Vietnam), whereas low pathogenic avian influenza virus (LPAIV) A/teal/Germany/Wv632/2005 (H5N1), and APMV-8, strain APMV-8/goose/Delaware/1053/76 (Acc. no. FJ619036) were obtained from the National Reference Laboratories for Avian Influenza (T. Harder) and Newcastle Disease at the Friedrich-Loeffler-Institut. NDV strain Herts33/56 was kindly provided by MSD Animal Health.

### Construction, transfection and recovery of chimeric NDV

The full length cDNA clone NDVH5Vlp is analogous to the previously described NDVH5Vm [34], but contains alterations in the NDV backbone [33]. In addition, the polybasic cleavage site (ERRKKRG) of H5Vm was altered to a monobasic (ETRG) one by site-directed mutagenesis with primers MPcleavlpF (5’-cag aaa tag ccc tca aag aga gac gag agg att att tgg agc tat agc-3’) and MPcleavelpR (5’-gct ata gct cca aat aat cct ctc gtc tct ctt tga ggg cta ttt ctg-3’). The resulting plasmid NDVH5Vlp was used for substitution of the open reading frames (orf) encoding NDV surface glycoproteins F and HN by the respective APMV-8 genes. In detail, a plasmid containing the 8,568 bp ApaI*-*BsiWI fragment of NDVH5Vlp was altered by Phusion polymerase chain reaction (P-PCR) (Finnzymes Phusion®, New England Biolabs®) [35] using megaprimers generated by reverse transcriptase-PCR (RT-PCR) of APMV-8 RNA and primers Ph8Forf_F (5’-ggt tgg cgc cct cca ggt gca aga tgg gta aaa tat caa tat atc taa tta ata gcg tg-3’) and Ph8Forf_R (5’-gga aac ctt cgt tcc tca tct gtg ttc aaa act tag att cac gtt tcg ttt gg-3’) for the F orf and Ph8HNorf_F (5’-gct tca ccg aca aca gtc ctc aat cat gag taa cat tgc atc cag ttt ag-3’) and Ph8HNorf_R (5’-cca act cct tta taa ttg act caa tca att att tct cac taa ttc ata caa c-3’) for the HN orf. Subsequently, the new 8,538 bp ApaI*-*BsiWI-fragment was inserted into plasmid NDVH5Vlp, resulting in chNDVFHN _PMV8_H5.

Transfection experiments were performed as described [36]. Virus was propagated in ECE after inoculation with 200 to 400 µl of transfection supernatant [11].

### RNA preparation, RT-PCR, and sequencing

Viral RNA was isolated from allantoic fluid using Trizol reagent (Invitrogen) according to the manufacturer’s instructions and virus identity was verified by RT-PCR using One-step RT-PCR Kit (Qiagen) to amplify selected regions. Virus identity was confirmed by sequencing (ABI).

### DNA sequencing, sequence assembly, and variant detection

Plasmid and viral RNA of chNDVFHN _PMV8_H5 were sequenced with the Genome sequencer FLX (GS FLX; Roche, Mannheim, Germany) using Titanium chemistry. For this purpose, cDNA was prepared from genomic RNA according to the GS FLX protocols for RNA library preparation. The sequencing libraries were generated automatically from cDNA or fragmented plasmid DNA using the SPRIworks Fragment Library System II (Beckman Coulter, Krefeld, Germany) and GS FLX rapid library adaptors. Raw data were assembled into complete sequences with the aid of the GS FLX assembler software newbler (v 2.6; Roche, Mannheim, Germany). To identify sequence variants possibly present in the population, raw sequencing reads were mapped along the complete sequences previously built from these raw data using the GS FLX reference mapper application (v 2.6; Roche).

### Kinetics of viral replication

Kinetics of replication were investigated in ECE after infection with 10^2.3^ TCID_50_ per egg followed by incubation at 37 °C and 55% humidity. Allantoic fluid was harvested at indicated time points and aliquots of two different pools were frozen at -70 °C for further analysis. Furthermore, viral replication was investigated in CEF and QM9 cells by infection of cells with a multiplicity of infection of 0.01 and titration of cell supernatants at different time points.

### Preparation of monospecific anti-F and anti-HN rabbit sera

For generation of recombinant vaccine viruses, the orfs encoding APMV8-F and APMV8-HN were amplified by PCR with primers Ph8F-orf_F and Ph8F-orf_R, or Ph8HN-orf_F and Ph8HN-orf_R using respective plasmids as templates. The ~1.7 kbp amplicons were purified, phosphorylated at their 5’ ends and cloned into the *Sma*I-digested transfer vector pCS43 which directs protein expression under control of the vaccinia virus p7.5K gene promoter. Vaccinia virus recombinants were generated as described [37], and tested for HN or F expression by indirect immunofluorescence reactions of infected cells with chicken serum against APMV-8. Two adult rabbits were infected with 1×10^8^ plaque forming units (PFU) of F- or HN-expressing vaccinia virus twice at a 2-week-interval. Sera were collected 2 weeks after the second immunization.

### Western Blot analyses

Virions which had been purified by ultracentrifugation in a continuous CsCl gradient (45%-20%) were separated by sodium dodecyl sulphate polyacrylamide gel electrophoresis (SDS-PAGE) and transferred to nitrocellulose membranes followed by immunostaining with monospecific, rabbit-α-H5 (AIV), rabbit-α-F (APMV-8), or rabbit-α-HN (APMV-8). After primary antibody incubation, binding of peroxidase-conjugated species-specific secondary antibody was detected by chemiluminescence substrate (Pierce) and visualized by ChemiDoc XRS+ (BioRad).

### Electron microscopy

Purified virions of the dialysed density gradient fraction were adsorbed to formvar coated nickel grids for 7 min. Grids were washed four times with phosphate buffered saline containing 0.5% bovine serum albumin (PB), and incubated with the respective antibodies, raised against AIV H5, APMV-8 F, and monoclonal mouse antibodies raised against NDV NP [38] for 45 min at room temperature. After several washings in PB, grids were incubated with protein A gold (10 nm, PAG 10, Biocell International), rabbit anti chicken gold, (10 nm, RCHL 10, Biocell International), or goat anti mouse gold (10 nm GAM 10, British Biocell International) for 45 min, followed by several washings with PB and finally by negative staining with phosphotungstic acid (pH 7.2).

### Determination of pathogenicity

The mean death time (MDT) was determined in 11-day-old ECE according to Hanson and Brandly [39]. The MDT is the mean time in hours for embryo mortality at the lowest dose that killed all embryos.

The intracerebral pathogenicity index (ICPI) was determined following European guidelines [40]. One-day-old SPF chickens were intracerebrally inoculated with 100 µl of 10^-1^ diluted virus stock (HA > 2^4^) and monitored for clinical signs and mortality for 8 days [41].

### Animal experiments

Vaccination and challenge experiments were carried out in BSL3 and BSL3+ experimental animal facilities of the Friedrich-Loeffler-Institut. All animal experiments were approved by the animal welfare committee (Landesamt für Landwirtschaft, Lebensmittelsicherheit und Fischerei Mecklenburg-Vorpommern, Thierfelderstraße 18, 18059 Rostock, LALLF M-V/TSD/7221.3-1.1-001/06, LALLF M-V/TSD/7221.3-1.1-053/10) and approved and supervised by the commissioner for animal welfare at the FLI representing the Institutional Animal Care and Use Committee (IACUC). Animals displaying severe clinical distress were sacrificed by exsanguination after knocking animals unconscious. Criteria for euthanasia were somnolence, apathy, akinesia or dyspnea. SPF chickens without maternally derived NDV antibodies (MDA-) hatched at the Friedrich-Loeffler-Institut were vaccinated oculonasally at 3 weeks of age with 0.1 ml of a virus suspension of chNDVFHN _PMV8_H5 containing 10^7^ TCID_50_/ml (10 chickens/vaccine; 4 non-vaccinated control chickens). Furthermore, chickens with maternally derived NDV antibodies (MDA+) were derived from a flock of SPF-white leghorn chickens, which had been immunized with a commercially available inactivated NDV vaccine (Nobilis® Newcavac, MSD Animal Health). Chickens were immunized either on day 1 or 7 after hatch (10-11 chickens/vaccine; 4 non vaccinated controls) following the same procedure as in MDA- animals. For comparison, twelve MDA+ chickens were oculonasally vaccinated with 10^7^ EID_50_/animal of recombinant NDVH5Vm on day 1 after hatch. To monitor vaccine virus shedding, oropharyngeal and cloacal swabs of MDA- chickens were taken on 2, 4, 6 and 12 days post vaccination (dpv). For the experiment with MDA+ chickens, sampling after vaccination was limited to combined oropharyngeal and cloacal swabs taken 4 dpv. Virus transmission was analyzed by introducing naive chickens 2 dpv which were subsequently tested for virus shedding on days 3 and 4.

Three weeks after vaccination, animals were challenged by inoculation via the oculonasal route with 10^6^ EID_50_/animal of HPAIV A/duck/Vietnam/TG24-01/05 diluted in 0.85% NaCl. An aliquot of each diluted virus batch was re-titrated to ensure proper dosage of the challenge virus. Clinical signs were evaluated (0 = healthy; 1 = sick; 2 = dead) over a period of 14 days and scored (clinical score) analogous to ICPI determination. Combined oropharyngeal and cloacal swabs were taken at 2, 4, 6 and 10 days post challenge (dpch). Heparinised blood samples were obtained from all animals before vaccination, before challenge infection, and from all surviving birds at the end of the observation period, and were tested for AIV-, NDV- and APMV-8- specific antibodies using the hemagglutination inhibition (HI) assay.

### Serology

Hemagglutination inhibition assay was done according to standard protocol [40]. Antibodies against AIV were also detected using the indirect ELISA ID Screen**®** Influenza A NP Antibody Competition ELISA kit (ID.Vet, Montpellier, France) according to the manufacturer’s recommendations.

### Titration of infectious particles and detection of viral genome

Titrations of swabs collected after vaccination and challenge infection were done in triplicate in microwell plates. Briefly, 50 μl of tenfold serial virus dilutions from 1:2 to 1:2,000 were added to 10^4^ LMH cells per well, cultivated without serum but adding TPCK trypsin (2 µg/ml) (Sigma) to the medium. The TCID_50_/ml was determined by observation of cytopathic effect (CPE) after 72 h of incubation with giant cell formation for the chimeric NDV and cell necrosis of the entire well for HPAIV.

Viral RNA was isolated by Nucleospin 96 Virus Core Kit (Macherey-Nagel) and QIAamp Viral RNA Mini Kit (Qiagen). AIV and NDV RNA was detected by RT-qPCR as described [34] and transformed to genome equivalents (GEQ) using calibration curves of defined RNA standards that were included with each RT-qPCR run. Standards were prepared as RNA run-off transcripts of the cloned NP-specific target fragment and used as a copy-based standard. The T7 RiboMAX™ Express Large Scale RNA Production System (Promega GmbH, Mannheim, Germany) was used for *in vitro* transcription of RNA after linearizing the NP gene containing plasmid by restriction digestion. RNA copies were calculated according to the formula: RNA molecules (per µl) = RNA concentration [µg/µl]/transcript length [nucleotides] x 182.5 x 10^13^.

### Statistical Data Analysis

Differences between groups were statistically tested by appropriate Wilcoxon-tests for paired or unpaired data, respectively. Bonferroni correction was applied in case of multiple testing. The global significance level was always 0.05. All calculations were performed using R software [42], Version 2.13.0 (2011-04-13).

### Histopathology and Immunohistopathology

On days 2, 4, 6 and 20 after vaccination of MDA- chickens, two animals each were euthanized and inspected for gross lesions. Tissue samples of brain, heart, liver, trachea, lung with air sac, spleen, bursa, thymus, proventriculus, gizzard (including peripheral nerves and ganglia), duodenum, pancreas, caecal tonsil, cloaca and Meckel's diverticulum were fixed with 4% phosphate buffered neutral formaldehyde and embedded in paraffin. Tissue sections (4 µm) were dewaxed and stained with hematoxylin and eosin. Furthermore, distribution of chNDVFHN _PMV8_H5 was assayed by immunohistochemistry with the avidin-biotin-complex (ABC) method [43] using antibodies against APMV-8 F, HPAIV H5, NDV M, and NDV NP diluted in Tris-buffered saline (0.1 M Tris base, 0.9% NaCl, pH 7.6) followed by biotinylated secondary antibody (Vector, Burlingame, CA; diluted 1:200 in Tris-buffered saline). A bright red signal was produced with an immunoperoxidase kit (Vectastain Elite ABC Kit, Vector), and the substrate 3-amino-9-ethylcarbazole (DAKO AEC substrate-chromogen system, Dako, Carpinteria, CA). Sections were counterstained with Mayer’s hematoxylin and sealed with aqueous medium (Aquatex, Merck, Darmstadt, Germany). Positive control sections of infected cultured cells were included, and rabbit serum against AIV NP (1:750) served as irrelevant control antibody.

## Results

### Generation of a chimeric NDV expressing F and HN of APMV-8 and H5 of AIV

**Figure 1:**
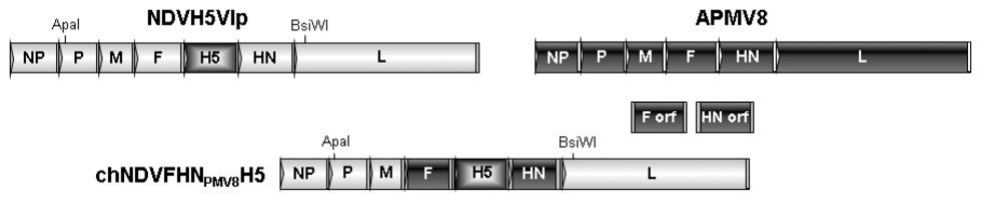
Construction of chNDVFHN _PMV8_H5. Schematic representation of plasmid construction: The full length plasmid NDVH5Vlp was cleaved by *Apa*I and *BsiW*I and the resulting 8,568 nt fragment was inserted into a pUC18 derivative. This plasmid was altered by Phusion PCR using megaprimers coding for the F orf, HN orf of APMV-8, respectively to substitute the F- and HN orfs of NDV by those of APMV-8. Afterwards, the resulting plasmid DNA was cut with *Apa*I and *BsiW*I and the 8,568nt fragment of the full length genome NDVH5Vlp was substituted by the modified *Apa*I and *BsiW*I-fragment (8,538 nt) sites, resulting in chNDVFHN _PMV8_H5.

Plasmid NDVH5Vm [33,34] was modified to generate chNDVFHN _PMV8_H5 by reverse genetics [11]. In both constructs H5 was inserted between F and HN, resulting in good expression levels of H5. A more 3’-proximal insertion of the foreign gene did not result in a higher expression level as previously shown [44]. Open reading frames of the surface proteins F and HN were exchanged by those encoding F- and HN- proteins of APMV-8 (amino acid identities are 44% for F and 37% for HN) using Phusion PCR with megaprimers (Figure 1). Recombinant virus was recovered after cotransfection of the full length plasmid with support plasmids pCiteNP, pCiteP and pCiteL into BSR T7 cells. Virus recovery was monitored by indirect immunofluorescence using monospecific sera against AIV H5. After propagation in ECE, virus identity was initially determined by amplification and sequencing of selected genome regions and confirmed by full genome sequencing.

### Expression and virion incorporation of the foreign proteins of chNDVFHN_PMV8_H5

**Figure 2:**
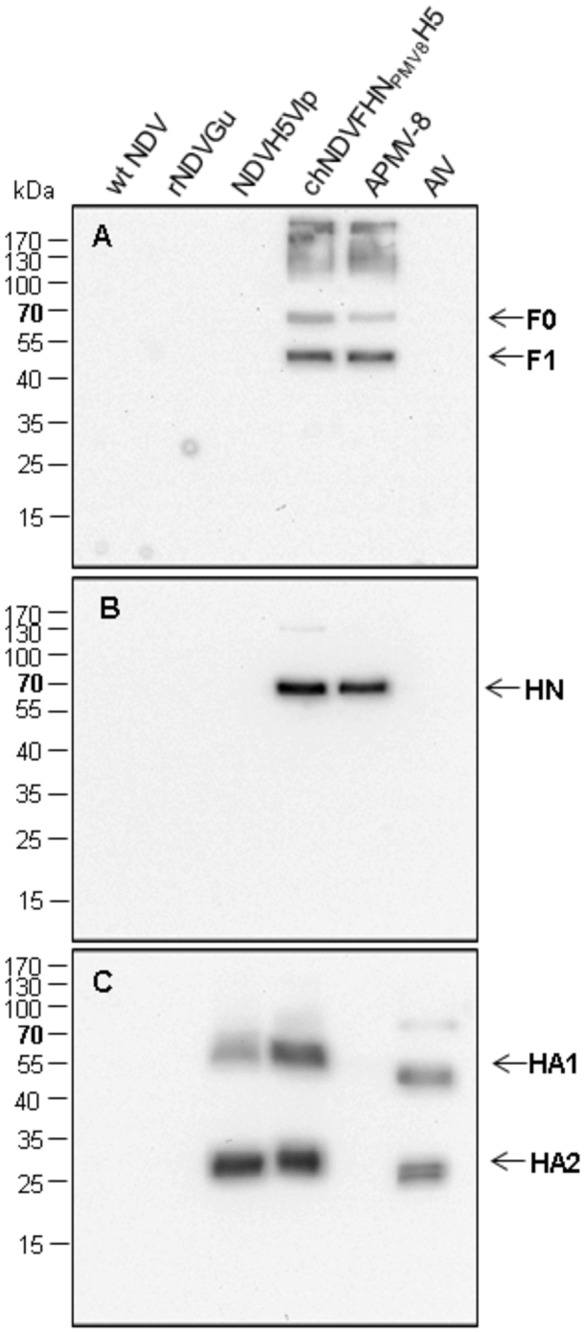
Protein expression and incorporation. After Western blotting of purified virion lysates, envelope proteins were visualized by immunostaining with rabbit-α-APMV-8 F (A), rabbit-α-APMV-8 HN (B) and rabbit-α-AIV H5 (C). After incubation with the respective primary antibody, binding of peroxidase-conjugated species-specific secondary antibody was detected by chemiluminescence substrate (Pierce). Identified proteins are indicated on the right, molecular weights of marker proteins (PAGE Ruler™ Prestained Protein ladder (Fermentas)) are indicated on the left.

**Figure 3:**
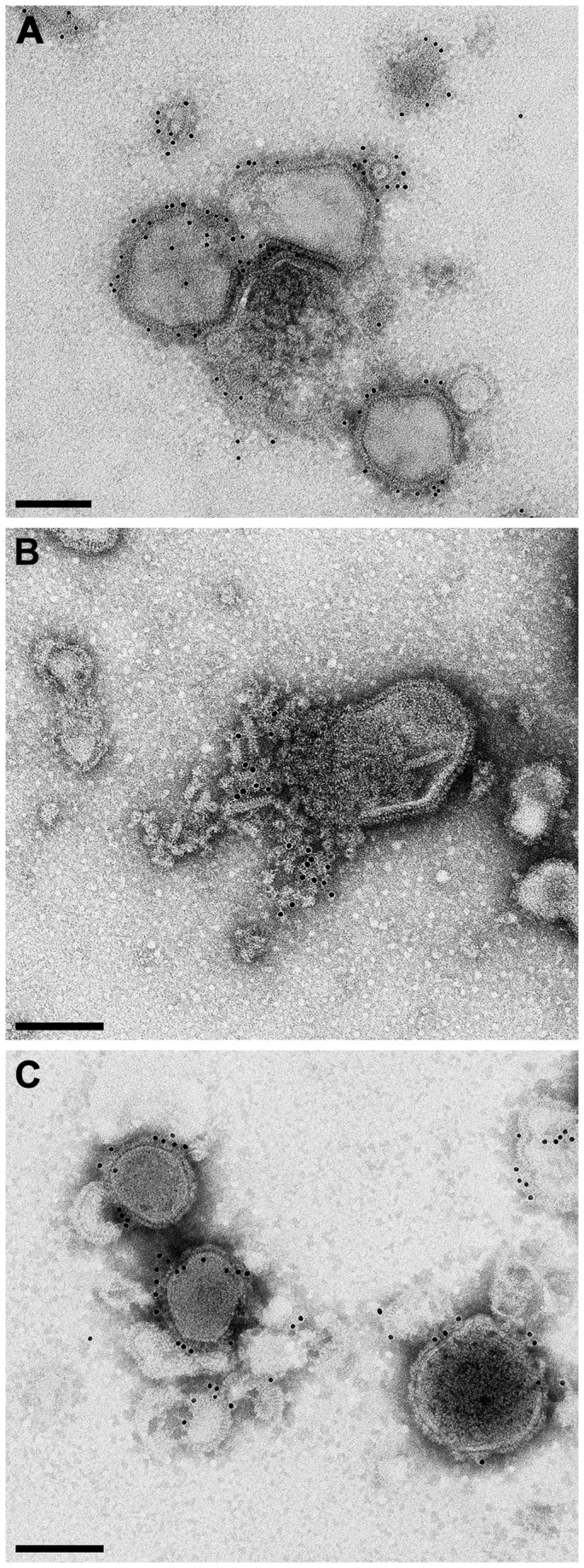
Immunoelectron microscopy of purified virions. Immunolabeling of chNDVFHN _PMV8_H5 was performed using a serum against APMV-8 F (A), a monoclonal antibody against NDV NP (B), or a serum against AIV H5 (C), followed by gold-tagged secondary antibodies. Scale bars: 150 nm.

Expression of the three foreign proteins and their incorporation into virions of chNDVFHN _PMV8_H5 was examined by Western blot analyses of purified virions (Figure 2) using monospecific rabbit sera against APMV-8 F, APMV-8 HN, and HPAIV H5. APMV-8-F and -HN proteins were detected with a molecular mass of about 60 kDa (F0 precursor), 45 kDa (F1 subunit; Figure 2A), and 65 kDa (HN; Figure 2B). The F2 subunit could not be detected due to its smaller size or lack of reactivity of the antiserum. HPAIV H5 was detected as cleaved ~50 kDa (HA1) and ~25 kDa (HA2) subunits (Figure 2C). For biosafety reasons, a low pathogenic influenza H5N1 was used as control, which explains the size differences between HA1 and HA2 proteins of chimeric NDV and low pathogenic AIV. The detection of APMV-8-F as well as of APMV-8-HN in purified virions indicates their incorporation into the viral envelope which was confirmed by immunoelectron microscopy. The envelope of intact virions was labeled by the APMV-8 F and HPAIV H5 specific antisera (Figure 3A, C), whereas only damaged virions exhibited labeling with an NDV-NP specific monoclonal antibody (Figure 3B). The APMV-8 HN-specific antiserum did not react in this assay. Taken together, these results clearly demonstrate incorporation of the foreign proteins APMV-8 F and HPAIV H5 into the viral envelope, whereas NDV NP is contained inside the virus particle, most likely within the ribonucleoprotein complex.

### chNDVFHN _PMV8_H5 replicates efficiently

**Figure 4:**
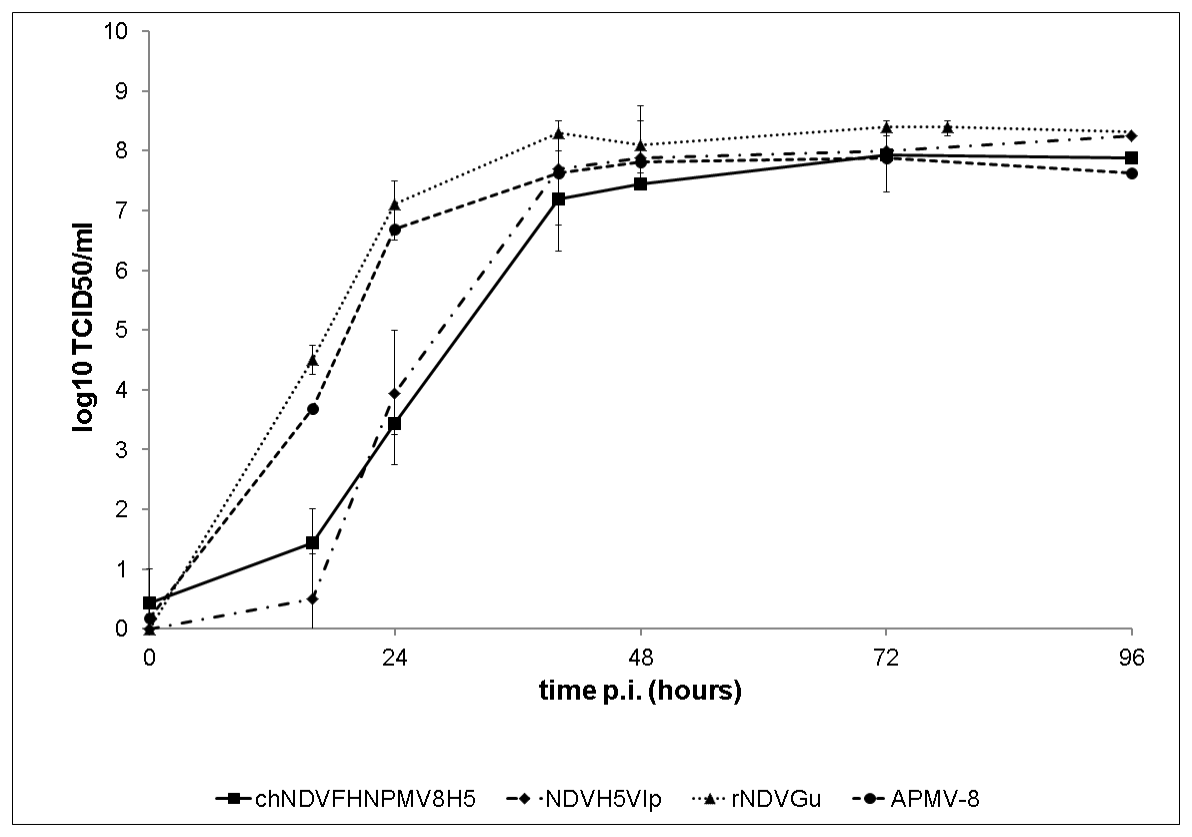
Kinetics of replication in ECE. Eggs were infected with 200 µl of a 10^3^ TCID_50_/ml dilution of chNDVFHN _PMV8_H5, APMV-8, NDVH5Vlp, and rNDVGu, respectively. Allantoic fluids were harvested at indicated time points after inoculation. Titers of progeny virus were determined by titration on QM9 cells, followed by indirect immunofluorescence.

Replication kinetics of chNDVFHN _PMV8_H5, the parental viruses NDVH5Vlp, and APMV-8, as well as recombinant NDV (rNDVGu) which does not express any foreign protein, were investigated in ECE (Figure 4). Both viruses carrying the AIV H5 gene exhibited a slightly reduced replication within the first 40 h after infection, but titers of all viruses were comparable at 48 h after infection and final titers reached about 10^8^ TCID_50_/ml, demonstrating that chNDVFHN _PMV8_H5 replication in ECE is as efficient as performance of the parental viruses. In CEF, chNDVFHN _PMV8_H5 replicates comparable to APMV-8, reaching a final titer of about 10^4^ TCID_50_/ml but not as well as the parental strain rNDVGu (final titer 10^6^ TCID_50_/ml). All investigated viruses, as expected, did not replicate in quail muscle cells (QM9) because of absence of trypsin-like proteases (data not shown).

chNDVFHN _PMV8_H5 was passaged ten times on ECE, resulting in virus stocks with comparably high titers (about 10^8^ TCID_50_/ml in 3^rd^ and 10^th^ passage). Titers were determined using three different antibodies (against AIV H5 as well as against APMV-8 F and NDV NP) for indirect immunofluorescence after titration yielding comparable values. This demonstrates continuing co-expression of the transgenes and the resident NDV gene indicating a highly stable expression of all genes of the new chimeric virus.

### Determination of virulence

An important characteristic of a vaccine virus is its low pathogenicity. The ICPI and MDT are determined to assess virulence of NDV. Low pathogenic NDV exhibit an ICPI < 0.7 and a MDT > 90 h, whereas the highest possible ICPI of 2.0 and a MDT lower than 60 h is typical for highly virulent, velogenic NDV. Determination of the ICPI of chNDVFHN _PMV8_H5 yielded a value of 0.29 and the MDT was > 168 h, demonstrating a lentogenic pathotype of the virus.

### Replication of NDVH5Vm in SPF chickens with and without NDV-specific maternally derived antibodies

**Figure 5:**
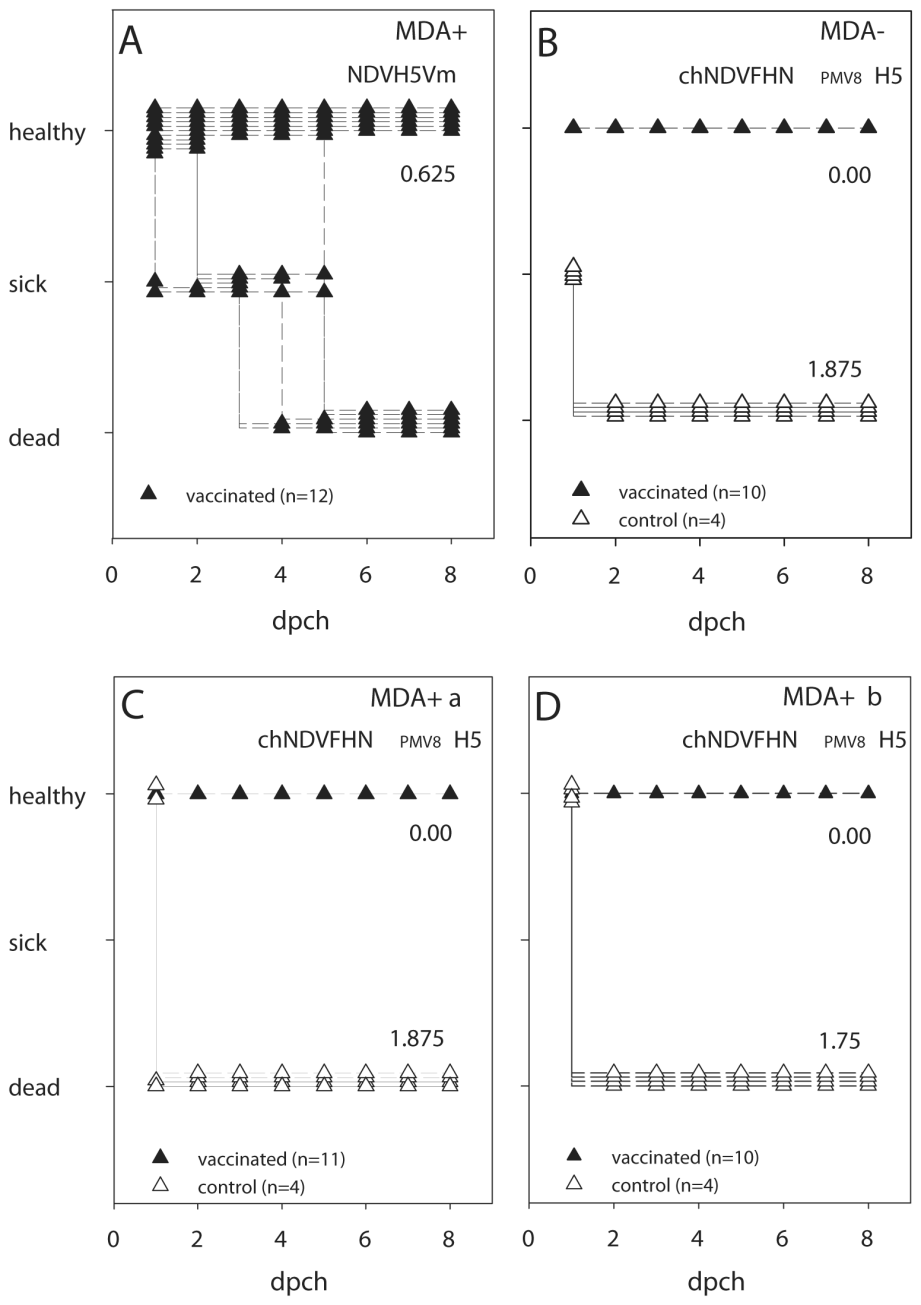
Clinical course after HPAIV H5N1 challenge infection. MDA+ chickens which were vaccinated with NDVH5Vm (A), and MDA- (B) as well as MDA+ birds (group a vaccinated on day 1 after hatch (C), group b vaccinated on day 7 after hatch (D)) which were vaccinated with chNDVFHN _PMV8_H5, and naive controls were challenged at day 21 after vaccination with A/duck/Vietnam/TG24-01/05 and daily classified as healthy (0), sick (1), or dead (2) over a period of 8 days. The average scores of all animals of each group are indicated.

The previously described recombinant vectored vaccine NDVH5Vm provided excellent protection in SPF chickens in the absence of maternally derived NDV specific antibodies (MDA-) as has been documented by our group [34]. However, protection rate was insufficient in MDA+ chickens. After immunization of one-day-old MDA+ chickens with NDVH5Vm (10^7^ EID_50_/animal) only 6 out of 12 chickens survived a challenge infection with homologous HPAIV (10^6^ EID_50_/ml) three weeks later (Figure 5A). Since NDV vaccination is widely practiced, and may even be compulsory in many countries, an improvement of the vector vaccine was considered mandatory for flexible use.

### Replication of chNDVFHN _PMV8_H5 in MDA- chickens

**Figure 6:**
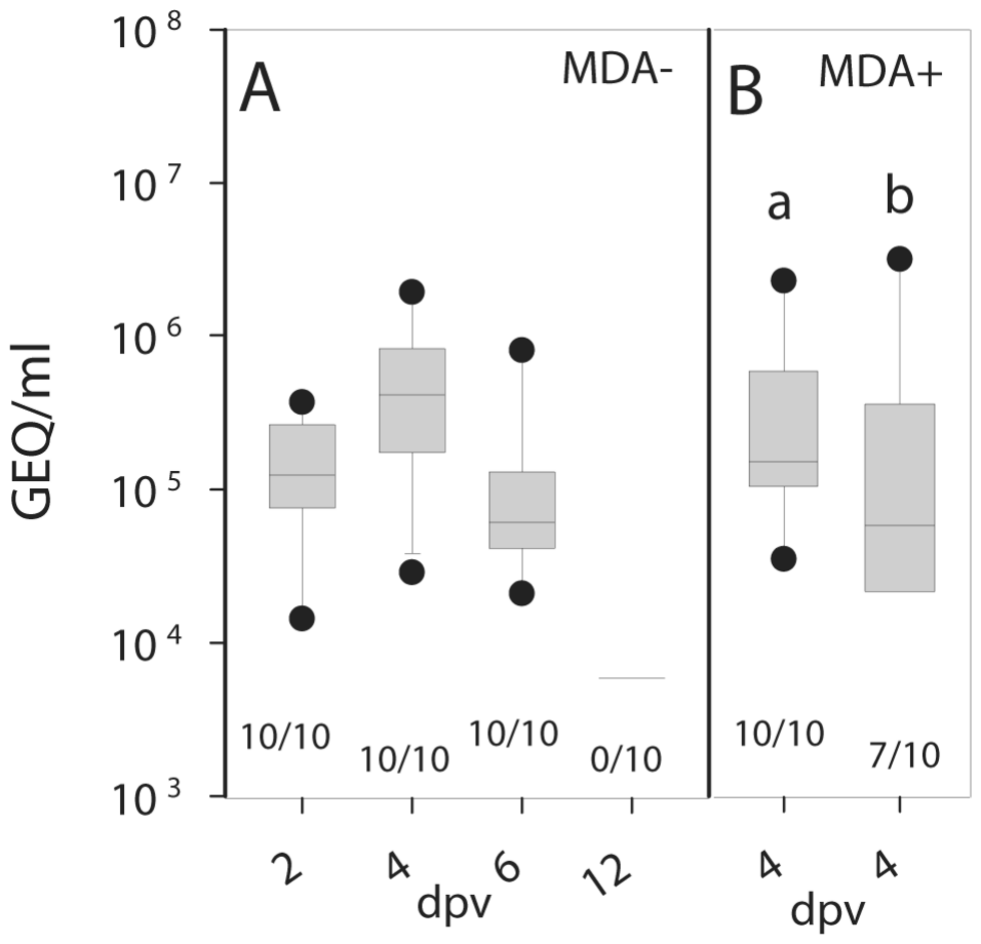
Virus shedding after vaccination. Swab samples taken on the indicated days from oropharynx of MDA- chickens (A) immunized with chNDVFHN _PMV8_H5 at three weeks of age and swabs combined from oropharynx and cloaca of MDA+ chickens (B) immunized on day 1 (group a) or day 7 (group b) after hatch were analyzed for the presence of NDV NP gene-specific RNA by RT-qPCR. Values were transformed to genome equivalents (GEQ) using calibration curves of defined RNA standards that were included with each RT-qPCR run. The number of positive swabs by RT-qPCR is given below the box plots. Significant differences (P < 0.0125, Bonferroni correction) between vaccinated groups and controls are indicated (*).

To analyze replication *in vivo*, three-week-old chickens were inoculated oculonasally with chNDVFHN _PMV8_H5. Presence of viral RNA (vRNA) could be demonstrated by reverse transcriptase-quantitative PCR (RT-qPCR) in oropharyngeal swabs peaking on 4 dpv (Figure 6A). Interestingly, infectious virus was detected by titration on LMH cells only in oropharyngeal swabs (2 of 10 animals at 2 dpv and 10 out of 10 animals at 4 dpv) with titers ranging between 7.11 x 10^1^ and 4 x 10^2^ TCID_50_/ml (Table 1). In contrast, infectious virus was not detected in cloacal swabs, whereas vRNA was detected up to 6 dpv in these samples (data not shown). Furthermore, in two naive sentinel animals that were added 2 dpv and monitored up to 6 dpv, no viral infectivity or vRNA was detected in oropharyngeal or cloacal swabs suggesting that chNDVFHN _PMV8_H5 was not transmitted from vaccinated to non-vaccinated chickens.

**Table 1:** Shedding of infectious virus after vaccination (TCID_50_/ml).

	**MDA-**								**MDA+ a**	**MDA+ b**
dpv	oropharyngeal				cloacal				combined	
	2	4	6	12	2	4	6	12	4	4
chicken nr.										
1	-	1,26 x 10^2^	-	-	-	-	-	-	-	-
2	-	4,00 x 10^2^	-	-	-	-	-	-	-	-
3	-	1,26 x 10^2^	-	-	-	-	-	-	-	-
4	-	1,26 x 10^2^	-	-	-	-	-	-	-	-
5	1,26 x 10^2^	1,26 x 10^2^	-	-	-	-	-	-	-	-
6	-	1,26 x 10^2^	-	-	-	-	-	-	-	-
7	-	1,26 x 10^2^	-	-	-	-	-	-	-	-
8	7,11 x 10^1^	1,26 x 10^2^	-	-	-	-	-	-	-	-
9	-	1,26 x 10^2^	-	-	-	-	-	-	-	-
10	-	1,26 x 10^2^	-	-	-	-	-	-	-	-

- no CPE was detected (corresponds to ≤ 4 x 10^1^)

None of the inoculated animals showed any clinical signs during the three weeks observation period. Furthermore, histological examination post necropsy of two chickens each on 2, 4, 6 and 20 dpv did not reveal any lesions or inflammation nor could viral antigen be detected in any of the tissues investigated, indicating only a local infection in the upper respiratory tract.

**Figure 7:**
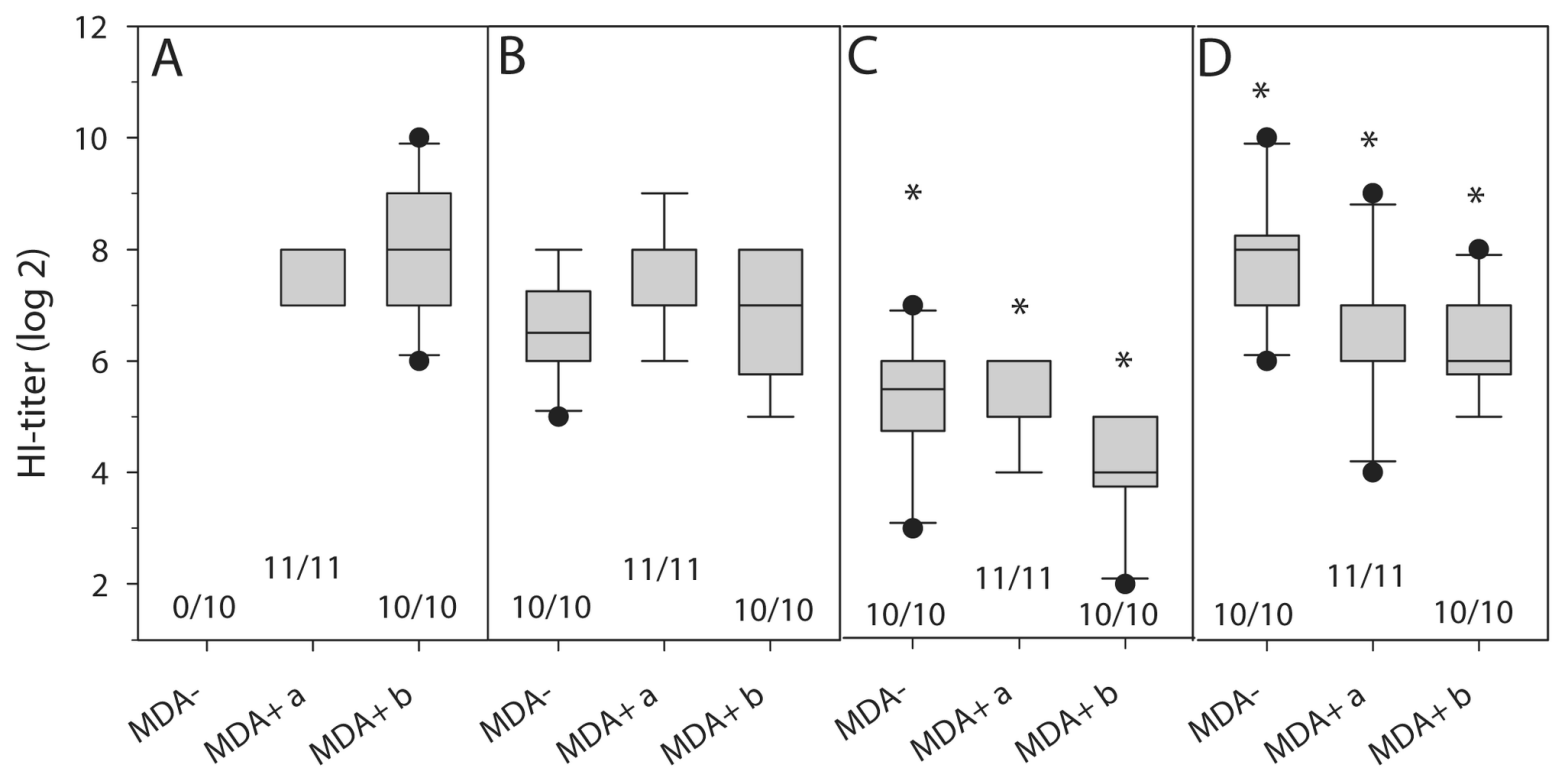
HI assay. Serum samples from immunized chickens before challenge collected at day 20 (MDA-) or 21 (MDA+) after vaccination with chNDVFHN _PMV8_H5 were tested for NDV (A), APMV-8 (B) and AIV H5 (C) specific antibodies by HI test. Furthermore, serum samples of MDA- chickens and MDA+ chickens vaccinated at day one (MDA+a) or day 7 (MDA+b) after hatch, taken 14 dpch were tested for AIV H5 (D) specific antibodies by HI assay. The number of animals considered seropositive (HI titer > 2 log_2_) is given below box plots. Significant differences (P < 0.05) between C and D are marked by *.

Antibodies against APMV-8 were already detectable by hemagglutination inhibition assay in all examined animals by 7 dpv, while AIV H5-specific antibodies were observed from day 14 pv. On 20 dpv, the day before challenge, HI titers against APMV-8 were detected on average 2^7.0^ and against AIV H5 2^4.9^ (Figure 7B, C). No NDV-specific antibodies were detected (Figure 7A), demonstrating lack of cross-reactivity between APMV-8 and NDV.

### chNDVFHN _PMV8_H5 protects MDA- chickens against lethal HPAIV infection

**Figure 8:**
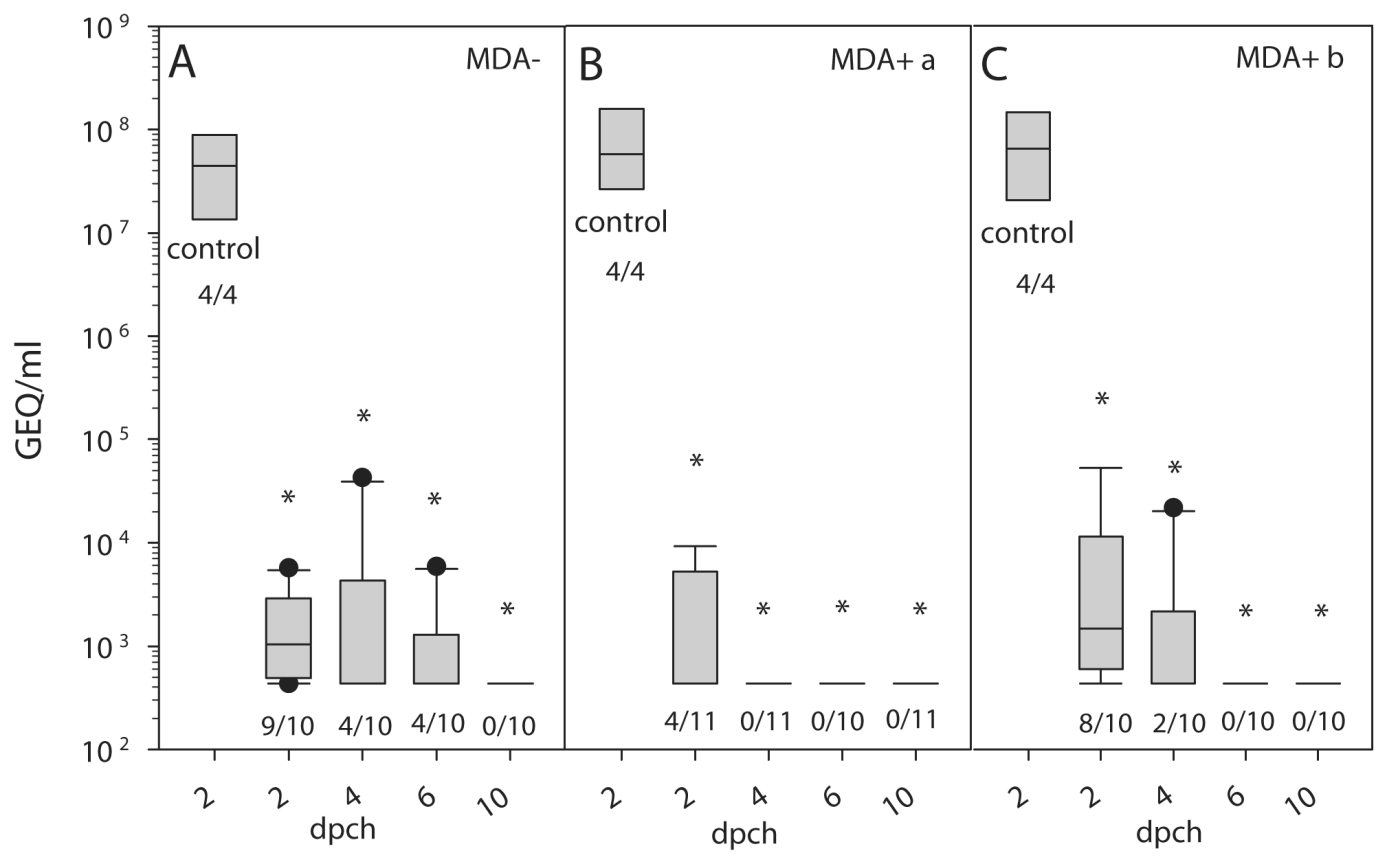
Virus shedding after challenge infection. Combined oropharyngeal and cloacal swabs of MDA- (A) and MDA+ chickens immunized on day 1 (group a (B)) or day 7 (group b (C)) were analyzed after challenge for presence of AIV NP gene-specific sequences by RT-qPCR. Values were transformed to genome equivalents (GEQ) using calibration curves of defined RNA standards that were included with each RT-qPCR run. The number of positive swabs by RT-qPCR is given below the box plots. Significant differences (P < 0.0125, Bonferroni correction) between vaccinated groups and controls are indicated (*).

To determine whether chNDVFHN _PMV8_H5 induces protection against HPAIV H5N1, ten vaccinated animals as well as a control group of four naive chickens were challenged oculonasally with 10^6^ EID_50_/animal. While all control animals died within two days after infection, all immunized chickens survived without exhibiting any clinical signs (Figure 5B). After challenge infection, AIV shedding was evident on day 2 by infectivity assay and by detection of vRNA (Figure 8A, Table 2) in vaccinated as well as in control birds. However, whereas only 5 of 10 vaccinated animals excreted between 1.26 x 10^2^ to 4 x 10^2^ TCID_50_/ml on day 2 pch, 3 of 10 animals shed between 7.11 x 10^2^ to 1.26 x 10^4^ TCID_50_/ml on day 4 pch but not subsequently. The non-vaccinated birds shed between 1.26 x 10^7^ to 1.26 x 10^8^ TCID_50_/ml (Table 2). Compared to the control group, vaccinated animals also shed significantly less vRNA, detected up to 6 dpch (Figure 8A). Thus, vaccination of SPF chickens without maternal antibodies with the chimeric recombinant resulted in full clinical protection and a drastic reduction in viral shedding.

**Table 2:** Shedding of infectious virus after challenge (TCID_50_/ml).

	**MDA-**					**MDA+ a**					**MDA+ b**				
dpch	Combined					combined					combined				
	2 (control)	2	4	6	10	2 (control)	2	4	6	10	2 (control)	2	4	6	10
chicken nr.															
1	1,26 x 10^8^	1,26 x 10^2^	-	-	-	1,26 x 10^7^	-	-	-	-	1,26 x 10^8^	1,26 x 10^3^	-	-	-
2	4,00 x 10^7^	4,00 x 10^2^	-	-	-	1,26 x 10^6^	-	-	-	-	1,26 x 10^8^	1,26 x 10^2^	-	-	-
3	4,00 x 10^7^	-	-	-	-	4,00 x 10^7^	-	-	-	-	1,26 x 10^7^	2,25 x 10^2^	-	-	-
4	1,26 x 10^7^	-	-	-	-	1,26 x 10^7^	2,25 x 10^3^	-	-	-	1,26 x 10^7^	1,26 x 10^2^	-	-	-
5		-	-	-	-		-	-	-	-		-	-	-	-
6		-	7,11 x 10^2^	-	-		-	-	-	-		2,25 x 10^2^	-	-	-
7		1,26 x 10^2^	-	-	-		2,25 x 10^3^	-	-	-		-	-	-	-
8		1,26 x 10^2^	2,25 x 10^3^	-	-		-	-	-	-		1,26 x 10^4^	-	-	-
9		1,26 x 10^2^	1,26 x 10^4^	-	-		-	-	-	-		2,25 x 10^2^	2,25 x 10^4^	-	-
10		-	-	-	-		2,25 x 10^2^	-	-	-		2,25 x 10^3^	-	-	-
11							1,26 x 10^3^	-	-	-					

- no CPE was detected (corresponds to ≤ 4 x 10^1^)

### chNDVFHN _PMV8_H5 protects MDA+ chickens against a lethal HPAIV infection

To analyze whether this vaccine is able to induce protection in offspring of NDV vaccinated animals, MDA+ chickens were vaccinated on day 1 (group a) or day 7 (group b) after hatch. On the day of hatch, yolk samples had NDV-antibody titers between 2^8^ and 2^11^ deliberately induced by prime-boost needle vaccination of layers. All chickens tolerated the immunization without showing any signs of disease regardless of the age at immunization. Combined oropharyngeal and cloacal swabs taken on 4 dpv were tested positive by RT-qPCR (Figure 6B) but not by infectivity assay (Table 1), again indicating a low level of vaccine virus replication.

On day 21 pv antibodies against APMV-8 as well as against AIV H5 were at comparable levels in MDA- and MDA+ animals (Figure 7B, C). At this point, NDV specific antibodies were also still detectable (2^7.5±0.5^ and 2^8.0±1.2^ in groups a and b, respectively) (Figure 7A) but were physiologically lower than levels obtained in yolk preparations on the day of hatch.

Upon challenge infection with HPAIV H5N1 three weeks after vaccination, all MDA+ chickens were protected from clinical disease, whereas all control animals died within 2 days (Figure 5C, D).

Non-vaccinated MDA+ control animals shed high amounts of virus on day 2 pch ranging from 1.26 x 10^6^ to 1.26 x 10^8^ TCID_50_/ml (Table 2) which were indistinguishable from MDA- control chickens. In contrast, only a fraction of the vaccinated animals shed virus on day 2 pch, at a strongly reduced level (Table 2). By 4 dpch, only swabs of two chickens vaccinated on day 7 were positive by RT-qPCR and one swab contained infectious virus (2.25 x 10^4^ TCID_50_/ml) (Figure 8B, C, Table 2). These results demonstrate that chNDVFHN _PMV8_H5 is capable to induce a high level of protection, not only in MDA- but also in MDA+ chickens.

### Differentiation between infected and vaccinated animals (DIVA)

**Figure 9:**
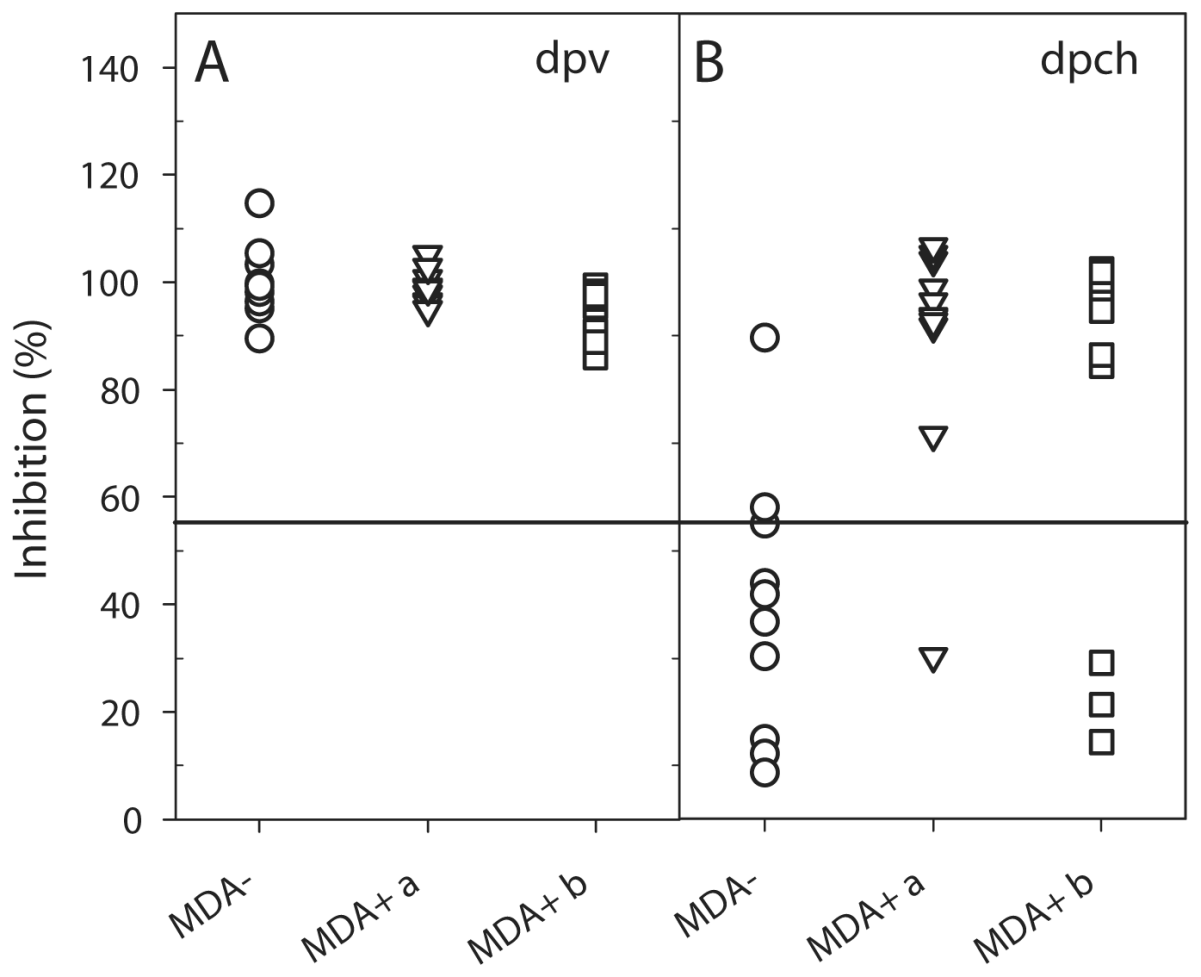
AIV NP-ELISA. Differentiation between vaccinated and infected animals (DIVA) based on antibodies against AIV NP. Sera of chickens collected 20 dpv (MDA-) or 21 dpv (MDA+) (A) and 14 dpch (B) were investigated by a competitive ELISA. Values ≤ 55% are indicative for the presence of AIV NP-specific antibodies.

Although AIV H5 specific antibody titers were significantly higher after HPAIV H5N1 challenge infection than after vaccination (Figure 7C, D), differentiation between vaccine-derived and infection-induced antibodies was impossible. However, the chNDVFHN _PMV8_H5 vector vaccine presents only the AIV HA, and testing for antibodies against other AIV proteins should allow differentiation (DIVA strategy). As expected, sera from chNDVFHN _PMV8_H5 vaccinated chickens were negative for AIV NP specific antibodies in ELISA (Figure 9), despite presence of AIV H5-specific antibodies (Figure 7C). After challenge infection, only 12 of the 31 vaccinated animals seroconverted to AIV NP-specific antibodies (Figure 9) suggesting that the limited replication of AIV challenge virus is not sufficient to induce a solid immune response toward the AIV NP.

## Discussion

Because of its versatility, NDV has been developed into a viral vector for heterologous proteins of unrelated infectious agents including AIV. These NDV-AIV vectored hybrid vaccine candidates have shown great promise in protecting chickens against NDV and AIV in situations where both are endemic. However, in emergency scenarios as occurred with the sudden advent of HPAIV H5N1, use of these vaccines is limited by the presence of anti-NDV antibodies derived from routine NDV vaccination of the target population. Thus, adaptation of the vector for this particular situation was necessary. We chose the generation of a chimeric NDV vector with surface proteins of a related, but antigenically different APMV to evade pre-existing NDV immunity allowing the vector virus to replicate and efficiently express the heterologous antigen.

The possibility of exchanging a functional protein by the equivalent polypeptide of a related virus was demonstrated already in the early 1990’s, e.g. by generation of a chimeric human and simian immunodeficiency virus [45]. Replacement of the structural protein genes of type 4 dengue virus by those of type 1 or 2 resulted in recombinant viruses with type 1 or 2 serological specificity but viral replication comparable with type 4 [46]. With the advent of reverse genetics also for negative stranded RNA viruses, targeted substitution of selected genes of these viruses became also possible [25]. The continuing scenario with numerous HPAIV infections especially in Asia and Africa which cause huge losses in poultry and represent a constant threat to humans, require the development of vaccines which are highly efficacious, easy to handle, and cost-efficient. NDV vector vaccines expressing HPAIV H5 or H7 have already been described as promising candidates for the development of such a vaccine [7,13,34]. However, the presence of maternally derived NDV-antibodies in many target populations may impair their replication in vaccinated animals and, therefore, may result in insufficient immunity. Thus, substitution of the surface glycoproteins F and HN by respective glycoproteins of another APMV subtype might be advantageous. Recently, functional substitution of F and HN of NDV by their counterparts of APMV-2 has been described [28]. We chose APMV-8 as donor because of low cross-reactivity between APMV-8 and NDV [30,31]. The generation of our NDV based recombinant expressing the two APMV-8 surface glycoproteins instead of the NDV counterparts demonstrated functional equivalence of the NDV and APMV-8 proteins. In addition, a third gene encoding a heterologous protein, HPAIV H5, inserted between the NDV F and HN genes, was also efficiently expressed and inserted into the viral envelope. Incorporation of H5 into the envelope seems to improve the vaccine virus quality [47]. Thus, concurrent expression of APMV-8 F and HN with HPAIV H5 and simultaneous incorporation into virions demonstrates the robustness of this system. A further advantage of this chimeric NDV is its potential as a marker virus, because of the possible DIVA strategy after an immunization with chNDVFHN _PMV8_H5. Furthermore, chNDVFHN _PMV8_H5 is of lentogenic pathotype, replicates as well as the respective NDV expressing the HPAIV H5 by reaching a final titer of about 10^8^ TCID_50_/ml, and stably expresses HPAIV H5 also after ten egg passages. These properties are a prerequisite for a well-tolerated and cost-effective vaccine. *In vivo*, after oculonasal inoculation of chickens no infectious virus was detected in combined oropharyngeal and cloacal swabs of MDA+ chickens, demonstrating a high level of safety. Furthermore, transmission of vaccine virus to naive sentinel animals was not observed. This indicates a low tendency of the live vaccine to spread to these highly susceptible animals and, therefore, most likely not to other species, which constitutes a prerequisite for a safe recombinant vaccine. However, considering the low number of sentinels, these results have to be confirmed and verified using different poultry as well as other species. In contrast, viral RNA was detected by RT-qPCR in swabs of MDA- as well MDA+ chickens (Figure 6A, B) which might be attributable to low-level local virus replication.

No clinical signs or local reactions were observed and immunohistochemistry failed to demonstrate viral antigen in internal organs including the trachea, lung, air sacs, and the gastrointestinal tract. This finding points to a low level of primarily local replication of chNDVFHN _PMV8_H5. Antibodies against APMV-8 were already detected at 7 dpv in MDA- chickens, whereas antibodies against HPAIV H5 were detected from 14 dpv. A solid immune response against both viruses was demonstrated three weeks after vaccination of MDA- as well as MDA+ chickens promising good protective efficacy. As expected, no cross-reactive immunity to NDV was observed by HI in MDA- chickens after vaccination with the chimeric vector virus which should eliminate interference with maternally derived NDV antibodies. This assumption was confirmed by vaccination of MDA+ chickens as early as one day after hatch. Like in MDA-, MDA+ vaccinated animals developed APMV-8 and AIV H5 specific antibodies and were fully protected against a lethal HPAIV H5N1 challenge. None of the MDA- or MDA+ vaccinated animals showed clinical signs after challenge infection, whereas all control animals died within 2 days, resulting in a clinical score of 1.75 to 1.875 (Figure 5B, C, D). This finding demonstrates the substantial benefit of the chimeric vector in protecting chickens with NDV specific - maternally derived antibodies. This level of protection could not be reached by the precursor NDVH5Vm. Analogous to clinical protection, only a fraction of vaccinated animals shed challenge virus and revealed detectable vRNA at a low level, whereas large amounts of vRNA as well as infectivity was detected in swabs of non-immunized control chickens (Figure 8 A, B, C, Table 2). However, vaccination with subsequent challenge boosted AIV H5-specific antibody responses in vaccinated chickens. In contrast, replication of challenge AIV was not sufficient to induce NP-specific antibodies in all animals within 14 days. Only 12 out of 31 animals sero-converted as detected by AIV-NP-specific ELISA. While our results do not exclude that chickens might have sero-converted later, they emphasize the difficulties immanent to serological DIVA strategies for epidemiological surveys. Employing a DIVA strategy is invariably negatively correlated to the potency of a vaccine. The stronger the protective efficacy of the vaccine in limitation or elimination of challenge virus replication, the weaker is the induction of immune responses by the challenge agent. An efficacious vaccine should suppress replication of a field virus, thereby reducing antigen stimulus and antibody response. These conditions highlight the need for active viral monitoring when AIV-vaccination is used.

If vaccination is considered to be part of an AIV prevention strategy, the described newly generated recombinant virus chNDVFHN _PMV8_H5 is a possible alternative vaccine candidate for a strategy using two different vaccines in breeders and in offspring. Using conventional inactivated AIV H5 vaccines in breeders, chicks could still be vaccinated at an early age using chNDVFHN _PMV8_H5 despite carrying NDV specific MDA. This would be of utmost importance, as broilers are the segment of poultry industry with highest numbers of animals. Mass application of a live vaccine would be a tremendous advantage for an AIV vaccination strategy. A disadvantage of the use of chNDVFHN _PMV8_H5 in comparison to NDVH5Vm is the loss of protection against ND. However, a combined vaccination with chNDVFHN _PMV8_H5 and NDV, both mass applicable vaccines, should be possible and is already under investigation. Furthermore, chNDVFHN _PMV8_H5 can be delivered successfully to very young chickens, which is of major importance for large scale vaccination. Further investigations have to demonstrate the impact of this vaccine on reducing spread of HPAIV H5 in AIV vaccinated populations. However, the presented vector vaccine platform would also provide an efficient tool for a fast adaptation of the vaccine to newly emerging AIV-variants by rapid exchange of the HA transgene.
